# Case report: severe cytomegalovirus primary infection in an immunocompetent adult with disseminated intravascular coagulation treated with valganciclovir

**DOI:** 10.1186/s12879-016-1343-3

**Published:** 2016-01-19

**Authors:** Niklas F. Müller, Matthias Schampera, Gerhard Jahn, Nisar P. Malek, Christoph P. Berg, Klaus Hamprecht

**Affiliations:** 1 Department of Internal Medicine I, University Hospital of Tuebingen, D-72076 Tuebingen, Germany; 2 Institute of Medical Virology and Epidemiology of Viral Diseases, University Hospital of Tuebingen, D-72076 Tuebingen, Germany

**Keywords:** CMV primary infection, Valganciclovir, DIC, Neutralisation, Avidity maturation

## Abstract

**Background:**

Disseminated intravascular coagulation (DIC) is a very rare complication of disseminated cytomegalovirus (CMV) infection. So far it is mainly described for immunocompromised patients.

**Case presentation:**

A 49-year-old immunocompetent Caucasian male presented with sudden onset of fever and DIC due to primary CMV infection, which was treated with Valganciclovir. CMV-specific IgG-avidity and epithelial cell-specific neutralisation-capacity developed five weeks after onset of symptoms. We describe the first case of an immunocompetent patient suffering from DIC due to a CMV primary infection successfully treated with Valganciclovir.

**Conclusions:**

Primary CMV infection can occur accompanied with life threatening complications even in immunocompetent patients. Immediate treatment with Valganciclovir should be considered as an early treatment of choice in severe cases since specific neutralisation capacity might need several weeks to develop.

**Electronic supplementary material:**

The online version of this article (doi:10.1186/s12879-016-1343-3) contains supplementary material, which is available to authorized users.

## Background

CMV infection is usually a threat in the haematological or transplant setting [1], since most of the patients with severe infections are immunocompromised, or during pregnancy or lactation in the context of congenital or postnatal CMV infection. Disseminated intravascular coagulation (DIC) due to CMV is reported more often in immunocompromised patients with severe CMV infections [2, 3], whereas in immunocompetent patients primary CMV infection is most frequently clinically silent [4]. So far there are only two published cases that suggest DIC due to CMV primary infection in immunocompetent patients [5, 6]. Here, we demonstrate for the first time the emergence of severe CMV primary infection-associated DIC. The diagnosis of CMV primary infection was established by detection of a high CMV IgM index and a low CMV IgG level without recgB1/2-IgG reactivity, and later confirmed by an increase in CMV IgG avidity maturation and epithelial cell-specific neutralisation capacity five weeks after the acute onset of fever episodes. The acute clinical manifestation, however, could apparently be controlled by initial short-term antiviral therapy with valganciclovir (VGCV).

## Case presentation

A 49-year-old Caucasian male appeared at the emergency room with fever, up to 40 °C, chills and dyspnea for one week. His medical record included hypertension, sleep apnoea, and hydronephrosis of the right kidney due to a former nephrolithiasis. He worked as a taxi driver and strongly denied any substance abuse. He neither reported any regular medication nor any signs of immunodeficiency in the past.

Upon admission the patient presented with blood pressure of 140/80 mmHg, heart rate of 113/min, body temperature of 39.2 °C, and a respiratory rate of 24/min. Physical examination yielded normal results. Initial blood tests revealed a normal white blood cell count (WBC) of 5.1 × 10^9^/l (4–9.5), an internationalised ratio (INR) of 1.5, an activated partial thromboplastin time (aPTT) of 42 s (<40 s), D-dimers of 15 μg/ml (0–0.23), a CRP of 21.02 mg/dl (<0.5), a procalcitonin level of 1.35 ng/ml (<0.1), and a lactate dehydrogenase activity of 823 U/l (<250). Liver and kidney function tests were within normal range. Abdominal ultrasound examination as well as chest X-ray images did not show abnormalities besides the known hydronephrosis. To look for a source of infection and to exclude thrombosis or pulmonary embolism, we performed a whole body CT-scan with contrast agent, but without gain of information. We started an empirical antibiotic treatment with piperacillin and tazobactam. However, fevers kept spiking within the following 24 h and the patient developed leukocytopenia (3.6 × 10^9^/l) and thrombocytopenia (66 × 10^9^/l, 150–450). Repeated blood cultures were negative but despite normal urine analysis, urine culture was positive for *Enterococcus faecium* (less than 10,000 CFU). Thus, diagnosis of urosepsis with DIC was most likely and antibiotic treatment was broadened to meropenem and vancomycin to include common resistant bacteria.

**Fig. 1:**
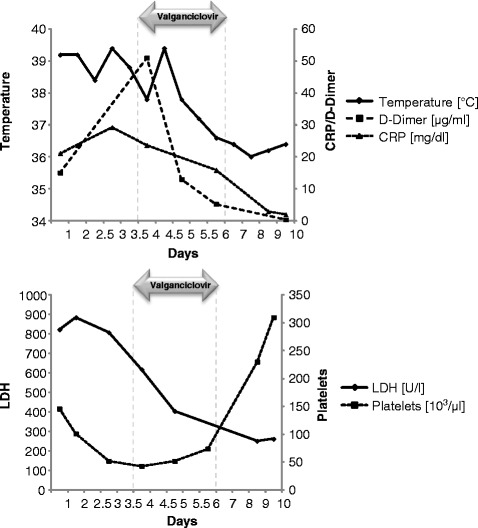
Monitoring of relevant blood tests in the course of infection. The arrow indicates the duration of valganciclovir therapy. Both diagrams show a return to normalcy of all parameters after brief antiviral therapy

Despite broad antibiotic therapy, in the following 24 h D-dimers and CRP increased and fevers were still ongoing. WBC and thrombocytes decreased further (Fig. 1). Given the clinical course of the disease with progressive leukocytopenia and thrombocytopenia, we suspected a viral infection and performed serological screening for tick-borne encephalitis, HHV-6, HIV, HBV, HCV, EBV, Parvovirus B-19, HSV-1/2, and CMV. While most serological tests were negative or only showed signs of past infection (for EBV and HSV-1/2), CMV-testing revealed an early primary CMV infection, characterised by a low IgG concentration and a high IgM index (>5.0) along with low IgG avidity (ECLIA, Roche) (Table 1). Disseminated CMV infection was confirmed by a positive PCR for CMV DNA in leucocytes and plasma. We therefore started valganciclovir treatment, with oral administration of 900 mg doses twice a day, due to the patient’s severe clinical conditions, in particular the worsened coagulopathy (Fig. 1). After administration of four valganciclovir doses, CRP and D-dimers decreased strongly, leukocyte and thrombocyte counts increased, and fever and chills disappeared (Fig. 1). We therefore stopped treatment with Valganciclovir after four doses. Further serological testing after four days of initiating antiviral therapy revealed still low avidity IgG antibodies to CMV; CMV-PCR was negative in leukocytes and plasma.

**Table 1:** Synopsis of CMV recombinant IgG epitope-specific avidity maturation (Mikrogen, Germany), CMV IgG increase, and decrease of IgM indices (ECLIA, Roche) in context of PCR results and viral shedding. In week 5, nested PCR was positive while real time PCR was negative, corresponding to viral copy numbers/ml plasma in the range of 200–600

Week	CMV IgG (U/ml; ECLIA) Cutoff ≥ 1,0	CMV IgG avidity (AI %, ECLIA)	recCMV IgG blot	recCMV IgG blot avidity	CMV IgM Index (ECLIA) Cutoff ≥ 1,0	recCMV IgM blot	EDTA blood PCR	Urine PCR (copies/ml)	Urine culture (NIEA pos Nuclei/ml)
0	9.46	3.13 (low)	IE1+++, p150+++, CM2++, p65+++, gB1/2 nd	IE1+, p150+, (low)	9.05	p150++	L+, P+		
1	9.07	3.26 (low)	IE1+++, p150+++, CM2+++, p65+++, gB1+/−	IE1+, p150+, CM2+/−	6.38	IE1+, p150++, CM2+, p65+	L-, P-	negative	
5	99.,79	41.67 (intermediate)	IE1+++, p150+++, CM2++, p65+++, gB1+, gB2 nd	IE1+, p150+, CM2+/−, (low)	2.16	p150++	L+, P+, <600 copies/ml (P)	2020	10and viral isolate
50	379.9	64.60 (high)	IE1+++, p150+++, CM2++, p65+++, gB1++, gB2+	IE1++, p150+++, CM2+, (high)	0.3	No reactivity	negative		

A follow-up in our outpatient clinic after one month showed the progress of CMV primary infection with an increase of CMV-specific IgG, avidity maturation and a decrease of IgM indices. CMV-DNA could be detected in plasma by nested PCR (LOD: 200 copies/ml) but not by quantitative real time PCR (LOD: 600 copies/ml). Therefore, the viral load ranged between 200 and 600 copies/ml of plasma. Viral shedding was observed in urine samples (Table 1) and throat swabs. About five weeks after the initial admission, the CMV specific antibodies had increased ten-fold, the avidity index had slowly increased to 35 %, which still represents low avidity, and IgM index had dropped from 3.2 to 1.4. Viral shedding could be documented with a viral isolate from urine microculture. Anti-CMV-gB2-IgG reactivity was not detectable, whereas gB1 reactivity was already weakly detectable (Table 1). The patient had no clinical symptoms of ongoing infection. Blood count, CRP, and clotting analysis were normal and he had resumed work.

In blood tests performed at a one year follow-up in our outpatient clinic, we could not detect anti-CMV-IgM antibodies, but we did detect high avidity IgG antibodies and results from plasma CMV-PCR were negative (Table 1). Blood count and clotting analysis had no pathological findings.

**Fig. 2:**
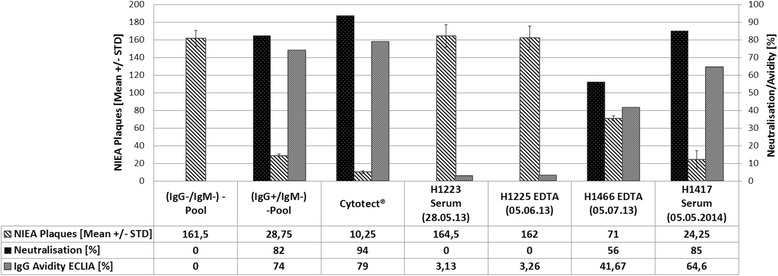
Epithelial cell-specific (ARPE-19) plaque reduction neutralisation capacity of the sera 1–4. Reference sera include a negative pool (*N* = 100 sera from seronegative women at birth), and a pool of latently CMV-infected women at birth (IgG positive, IgM negative, high IgG avidity) as well as the hyperimmunoglobulin preparation Cytotect (Biotest, Germany) [11]. The shaded columns show the mean number (*N* = 3 replica) of CMV IE1-stained plaques (N_IEA_ plaques) in the absence of CMV-specific antibodies after 5 days of incubation. Black columns show the percentage of neutralisation via plaque reduction of each serum panel. Gray columns show CMV IgG avidity (AI ECLIA, %). Specific NT capacity is not present in early sera of the patient (within one week of admission). However, after 5 weeks, NT_50_ values are reached

The follow-up tests performed show the typical dynamics of a primary CMV infection with a loss of p150 rec IgM reactivity (IgM index <1,0), a steep increase in CMV IgG (about 4-fold), and the emergence of gB2-IgG, which indicates CMV neutralising antibodies (Table 1, Fig. 2, Additional file 1). Interestingly, the CMV specific antibody pattern had already been present with high titers at the very onset of symptoms, while gB1 and gB2 reactivity only developed slowly (Fig. 2, Additional file 1). These findings support the initial diagnosis of an acute onset of a primary CMV infection.

Retrospectively performed longitudinal epithelial cell-specific neutralisation experiments revealed the emergence of neutralising antibodies about five weeks after onset of clinical symptoms. This emergence is accompanied by a steep increase of CMV avidity maturation. However, the neutralising capacity was not fully developed five weeks after onset of symptomatic infection (Fig. 2).

Details of the methodology can be found in Additional file 1 “Methods”.

## Conclusions

CMV is able to infect many different cells in various tissues in the human body. Several CMV in vitro assays have proven thrombogenic potential and a procoagulant phenotype of endothelial cells [7]. In rat models, CMV infection has led to haemorrhage, thrombosis, and abnormal coagulation blood tests due to infection of endothelial cells comparable to DIC [8]. A recent report also suggests that CMV activates and interacts with a subset of human platelets without immediate thrombotic events but with enhanced inflammation due to neutrophil extravasation and formation of platelet-leukocyte aggregates [9]. In contrast, thrombosis in both arterial and venous circulation, detected via anti-cardiolipin-and anti-ß (2) glycoprotein I- antibodies, has been described in an immunocompetent patient with primary CMV infection [10].

DIC appears to be a very rare complication of disseminated CMV infection in general. It has been described rarely for immunosuppressed patients [2, 3], and only twice for immunocompetent patients [5, 6]. The first patient, reported in 1972, died of severe CMV cardiomyopathy and DIC after being treated with heparin and digoxin [6]. The second patient, reported in 2006, survived severe DIC due to plasma exchange treatment [5]. Treatment with ganciclovir or valganciclovir was not conducted in the second patient. To the best of our knowledge, the case presented here is the first case in which an immunocompetent patient with disseminated intravascular coagulation was treated with short-term valganciclovir administration with longitudinal analysis of CMV IgG avidity maturation and epithelial cell-directed neutralisation during CMV primary infection.

Treatment of CMV infections in immunosuppressed patients regularly includes the reduction of immunosuppressants, and/or treatment with antiviral drugs such as ganciclovir or valganciclovir [1]. In immunocompetent patients, however, a primary CMV infection is mostly self-limiting. Besides treating the underlying cause of DIC, DIC is usually treated with antifibrinolytic drugs such as cyclocapronic acid, supplementation of clotting agents, or plasma and low dose heparin.

In our patient, the primary CMV infection was diagnosed after excluding other possible diagnoses (e.g., negative blood cultures, no improvement after broad antibiotic treatment) and by detection of CMV-specific IgM reactivity in the context of low IgG avidity and low IgG levels in the absence of anti-CMV recgB2 IgG with broad epitope specific IgM reactivity against CMV IE1, p150, CM2, and p65 [11]. Fibroblast-specific neutralising antibodies usually become detectable after 4–5 months during primary infection. In contrast, the epithelial cell–specific neutralisation already emerges within five weeks of the onset of clinical symptoms. Therefore, immune control can be achieved about five weeks after the onset of symptoms.

We decided to start the treatment with valganciclovir as an attempt to stop the ongoing fever spikes and the progressive coagulopathy and aiming to avoid plasmapheresis. According to other reports of immunocompetent patients suffering from CMV infection of other organs (e.g., lung or bowel), treatment with only a few doses of valganciclovir influenced the course of disease positively [12–15], and indeed, our patient improved rapidly within 24 h. Therefore, plasmapheresis and further treatment of DIC could be avoided. We suggest that inhibition of CMV proliferation with targeted antiviral treatment reduced the viral load significantly and stopped the progression of endothelial inflammation and therefore terminated the DIC. Thus, only immediate therapy with valganciclovir could successfully bridge the gap between the onset of symptoms and immune control.

## Consent

Written informed consent was obtained from the patient for publication of this Case report and any accompanying images. A copy of the written consent is available for review by the Editor of this journal.

## Additional file

Additional file 1: Methods. (DOCX 19 kb)

## Abbreviations

AI: avidity index (%)
CMV: specific recombinant antigens (IE1: immediate early antigen, p150 tegument phosphoprotein, CM2 fusion protein of UL44 (DNA polymerase accessory protein) and DNA binding protein UL57), p65 tegument phosphoprotein, gB1/2: glycoprotein B1/2-specific antigens)
CMV: cytomegalovirus
COI: cut-off index
DIC: disseminated intravascular coagulation
EBV: Epstein-Barr-Virus
ECLIA: electrochemiluminescence double antigen sandwich immunoassay
HBV: hepatitis B virus
HCV: hepatitis C Virus
HHV-6: human herpesvirus 6
HIV: human immunodeficiency virus
HSV-1 or 2: herpes simplex virus 1 or 2
L: leukocyte
LOD: limit of detection
N_IEA_: number of CMV immediate early positive fibroblast nuclei
P: plasma
PRNT: plaque-reduction neutralization assay
