# Supplementary material for: Case report: severe cytomegalovirus primary infection in an immunocompetent adult with disseminated intravascular coagulation treated with valganciclovir
Source: BMC Infect Dis. 2016 Jan 19;16:19. doi: 10.1186/s12879-016-1343-3 (PMC4719720; doi:10.1186/s12879-016-1343-3)
Supplement: Additional file 1: Methods. — (DOCX 19 kb) [file 12879_2016_1343_MOESM1_ESM.docx]

**Methods**

**CMV serology**

For determination of CMV IgG, IgM, and IgG avidity we used the Elecsys system from Roche Diagnostics, Mannheim, Germany, running on the automated Cobas e601/6000 system. For CMV IgG a double antigen sandwich assay with monomeric antigens labelled with Ruthenium and Biotin and streptavidin-coated magnetic-beads was used. Electrochemiluminescense read out in U/ml was negative < 0.5; borderline 0.5-1.0; positive ≥ 1.0. Elecsys IgM is a µ-capture assay with multimeric antigens. Read out: IgM negative < 0.7 COI (Cut-off index); borderline > 0.7, < 1.0 COI; positive: ≥ 1.0 COI. Read out for CMV IgG avidity assay (avidity index, AI %): AI <45, low; AI >45 <55, intermediate; AI ≥ 55, high.

**CMV neutralization**

We used as target cells human retinal pigment-epithelial cells (ARPE-19). The viral target strain H2497-11 originated from a primary viral isolate, propagated on ARPE-19 cells of amnion fluid of a mother with primary CMV infection. The serum samples of the DIC patient were heat-inactivated for 30 min at 56 °C, followed by centrifugation at 2700 × *g*. The reference sera included a CMV-specific hyperimmunglobulin (Cytotect®, Biotest), as well as two serum-pools (each N=100) from seropositive, latently infected (CMV IgG+/IgM-) and seronegative (CMV IgG-/IgM-) mothers at birth. Using a viral dilution of 1:800, we yielded about 150 isolated CMV-IEA stained (not confluencing) plaques in the seronegative control pool in a 5 day PRNT (plaque-reduction neutralization assay) [7]. Initial incubation of each 100 µl of viral predilution (1:400) and prediluted patient sera (1:800) was performed for 90 min at 37 °C. Thereafter, each 100 µl of the virion-antibody-mixture was inoculated on each 3 replica of ARPE-19 monolayers and incubated for 5 days at 37 °C in 200 µl DMEM-10% FCS, followed by fixation and CMV-IEA-immunoperoxidase-staining.

**Direct viral detection**

Virus isolation using human foreskin fibroblasts; primary human foreskin fibroblasts were derived from surgical circumcision of up to 2 year-old boys and were repeatedly propagated in microculture plates. DNA extraction and nested PCR (nPCR) of the CMV IE1-Ex4 region were performed as described previously [7]. The quantitative real time CMV PCR was performed using the CMV R-Gene PCR-kit (Argene, Biomerieux) with a limit of detection about 600 copies/ml, while nPCR has a limit of detection of about 200 copies/ml.

**Coagulation assays**

INR was determined using HemosIL RecombiPlasTin 2G reagent, Instrumentation Laboratory Company, Bedford, MA, USA. APTT was determined using HemosIL APTT-SP reagent, Instrumentation Laboratory Company, Bedford, MA, USA. Both assays were performed on an ACL Top, Instrumentation Laboratory Company, Bedford, MA, USA.
